# Antithrombotic therapy at discharge and prognosis in patients with chronic coronary syndrome and atrial fibrillation who underwent PCI: a real-world study

**DOI:** 10.1186/s12959-024-00628-1

**Published:** 2024-07-17

**Authors:** Yimeng Wang, Yanmin Yang, Lulu Wang, Han Zhang, Jiang-shan Tan, Yuyuan Shu

**Affiliations:** Emergency Center, State Key Laboratory of Cardiovascular Disease, National Center for Cardiovascular Diseases of China, Fuwai Hospital, Chinese Academy of Medical Sciences and Peking Union Medical College, 167 Beilishilu, Xicheng District, Beijing, China

**Keywords:** Atrial fibrillation, Chronic coronary syndrome, Antithrombotic therapy, Anticoagulant therapy

## Abstract

**Background:**

This study aimed to describe the status of antithrombotic therapy at discharge and prognosis in patients with atrial fibrillation (AF) and chronic coronary syndrome (CCS) who underwent percutaneous coronary intervention (PCI).

**Methods:**

This was an observational, prospective study. The primary endpoint was major adverse cardiovascular events (MACE), including all-cause death, myocardial infarction, stroke/transient ischemic attach (TIA), systemic embolism or ischemia-driven revascularization. Bleeding events were collected according to the Thrombolysis in Myocardial Infarction (TIMI) criteria.

**Results:**

Between 2017 and 2019, a cohort of 516 patients (mean age 66, [SD 9], of whom 18.4% were female) with AF and CCS who underwent PCI were evaluated, with a median followed-up time of 36 months (Interquartile range: 22–45). MACE events occurred in 13.0% of the patients, while the TIMI bleeding events were observed in 17.4%. Utilization of TAT (triple antithrombotic therapy) (*P* < 0.001) and oral anticoagulation (OAC) therapy (*P* < 0.001) increased through years. History of heart failure (HF) (Hazard ratio [HR], 1.744; 95% confidence interval [CI], 1.011–3.038) and TAT (HR, 2.708; 95%CI, 1.653–4.436) had independent associations with MACE events. OAC (HR, 10.378; 95%CI, 6.136–17.555) was identified as a risk factor for bleeding events. A higher creatine clearance (HR, 0.986; 95%CI, 0.974–0.997) was associated with a lower incidence of bleeding events.

**Conclusions:**

Antithrombotic therapy has been improved among patients with AF and CCS who underwent PCI these years. History of HF and TAT were independently associated with MACE events. Higher creatine clearance was protective factor of bleeding events, while OAC was a risk factor for TIMI bleeding events.

**Supplementary Information:**

The online version contains supplementary material available at 10.1186/s12959-024-00628-1.

## Introduction

A previous study demonstrated that coronary artery disease (CAD) is more prevalent among patients with atrial fibrillation (AF), ranging from 15 to 50% [1]. Chronic coronary syndrome (CCS) is a manifestation of CAD, often occurring concurrently with AF [2]. It may require percutaneous coronary intervention (PCI) to improve anginal symptoms or reduce the risk of subsequent myocardial infarction and death [3]. Regarding AF, anticoagulant therapy is crucial for reducing adverse outcome [4]. In terms of CCS post-interventional treatment, antiplatelet therapy, specifically double antiplatelet therapy (DAPT) consisting of aspirin and clopidogrel was recommended [5, 6]. Based on randomization clinical trials involving patients with CCS and AF undergoing PCI, updated guidelines suggest this population should receive triple antithrombotic therapy (TAT) at discharge [7]. The WOEST trial, which involved patients requiring OAC therapy after PCI and provided both DAT and TAT [8]. This trial revealed that DAT was associated with a lower incidence of bleeding episodes compared to TAT (HR 0.36, 95% CI 0.26–0.50, *P* < 0.001). Moreover, Asian patients have been observed to have a higher bleeding risk compared to other races [9, 10]. In the COMPASS analysis, Asians exhibited higher rates of intracranial haemorrhage (0.63% vs. 0.29%, *P* = 0.01) and minor bleeding (13.61% vs. 6.49%, *P* < 0.001) [11].

On the contrary, undertreatment with antithrombotic therapy might lead to ischemic stroke and recurrent PCI [12, 13]. Accordingly, to prevent patients from both ischemic and bleeding events, it is significant to find the balance between over- and under-treated antithrombotic regimen, especially for Asian patients. The AVIATOR 2 prospective registry revealed that TAT was prescribed in 66.5% of patients with AF and PCI, DAPT in 20.7% and dual antithrombotic therapy (DAT) in 12.8% [14]. Nevertheless, limited data was available in Asian populations. Therefore, our research aimed to describe the current status of antithrombotic therapy at discharge, focusing on Chinese patients with CCS and AF who underwent PCI, and identify underlying predictors affected prognosis.

## Methods

### Study design and participants

**Fig. 1:**
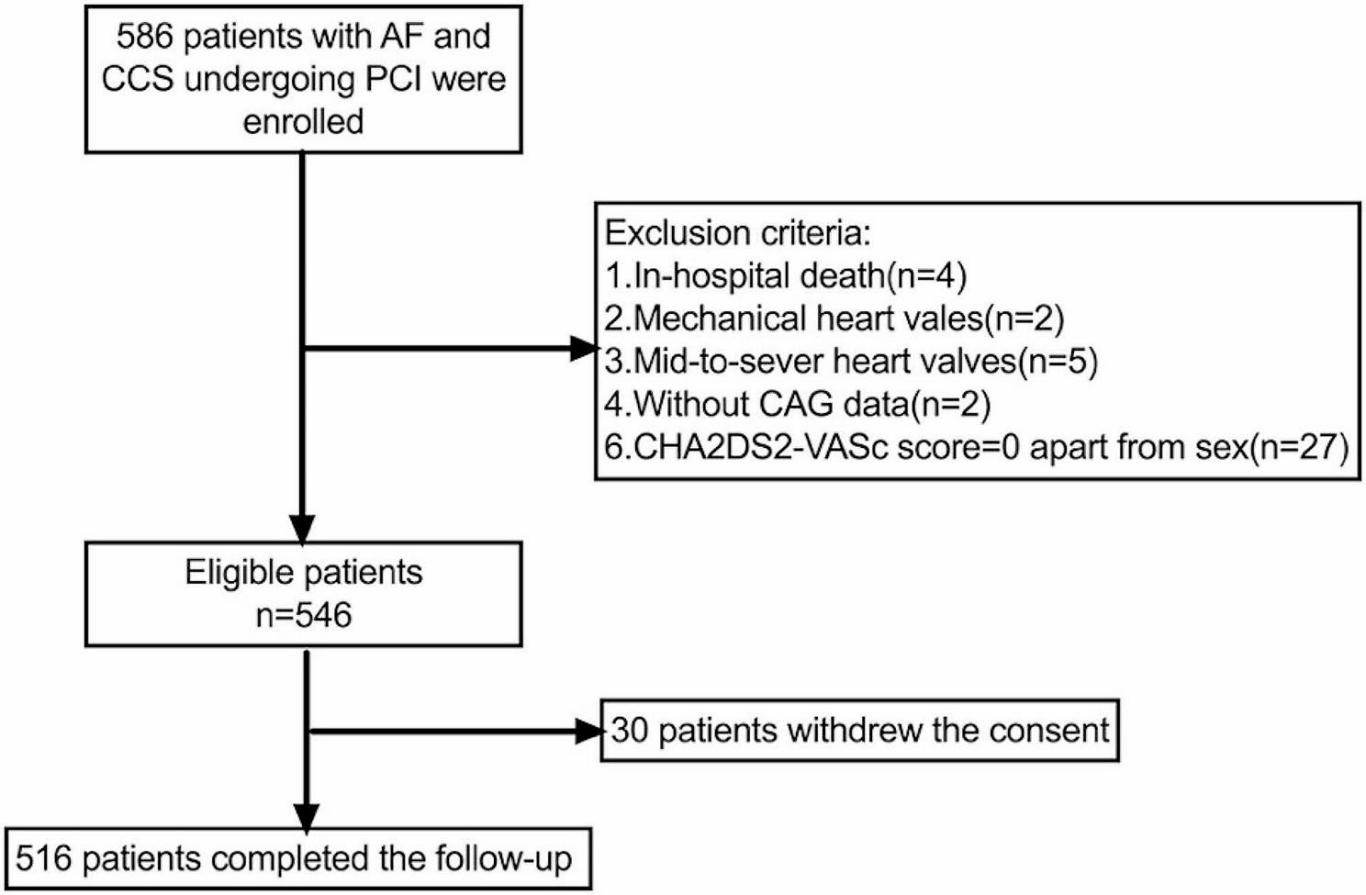
Flow chart of enrolled patients

This study was an observational, prospective, single center study of adults with AF and CCS who underwent percutaneous coronary invasive treatment from 2017 to 2019 in Fuwai Hospital, Beijing, China. Participants aged over 18 with AF and CCS who underwent invasive treatment were able to get eligibility. Patients who died in the hospital, discharged without coronary angiography (CAG) or antiplatelet therapy, or were diagnosed with mid-to-severe mitral stenosis or mechanical valves were excluded. A total of 546 participants were included, all of whom possessed at least one risk factor for stroke, aside from gender, as determined by the CHA2DS2-VASc score (Fig. 1). The study design and protocol have been approved by the Ethics Committee of Fuwai Hospital (Approved No. 2017 − 923) and conformed to the Declaration of Helsinki. All the patients had signed consent to participate in this study.

### Definitions

AF diagnosis relied on electrocardiography (ECG), holter monitoring, and clinical symptoms. CCS was characterized by the presence of coronary artery disease (CAD) confirmed through CAG and stable anginal symptoms [15]. Patients deemed at high risk of ischemic thromboembolism had a CHA2DS2-VASc score ≥ 1, excluding sex, while a high risk of bleeding was defined as HAS-BLED score ≥ 3 [16]. Major adverse cardiovascular events (MACE) were defined as composite endpoint of all-cause death, stroke or transient ischemic attach (TIA) or systemic embolism (SE), myocardial infarction (MI) or ischemic driven revascularization [17]. Bleeding events were recorded following the Thrombolysis in Myocardial Infarction (TIMI) criteria [8]. Definitions of medical history were provided in supplementary material.

### Antithrombotic regimens

Antithrombotic regimens were documented at the time of discharge, encompassing both antiplatelet and oral anticoagulant (OAC). The combination of two types of antiplatelet therapy and anticoagulant therapy was referred to as TAT. OAC combined with only one type of antiplatelet therapy was classified as DAT. OAC were further categorized into non-vitamin K oral anticoagulants (NOAC, e.g. rivaroxaban) and warfarin (vitamin K anticoagulants).

### Data collection

Data collection included demographic information, prior comorbidities, and medication usage at discharge. The median follow-up time was 36 months (Interquartile range :22–45). The primary outcome was major adverse cardiovascular events (MACE), defined as a composite of all-cause death, stroke or TIA or SE, MI or ischemic driven revascularization. Bleeding events were assessed using the TIMI criteria. Risk scores were calculated by their treating physicians and confirmed by computer using medical records. The follow-up outcomes were recorded at 1, 6 and 12 months after index events and then annually until the end of 2021. This information was gathered through telephone calls, re-hospitalization, and outpatient visits facilitated by trained research personnel. During the follow-up period, 30 patients withdrew the consent.

### Statistical analysis

Continuous variables were presented as mean ± standard deviation (SD) or as median with lower and upper quartiles and tested by using ANOVA test, Kruskal-Wallis *H* test, Mann-Whitney *U* test or *t* test, while categorical variables are presented as counts and percentages and tested with χ^2^ test. A multiple logistic regression analysis was performed to appraisal the independent factors which were able to predict the prescription of OAC, with odds ratios (OR) and 95% confidence interval (95% CI). Multivariate Cox proportional-hazards model was used to adjust confounding factors and found out factors associated with MACE events and TIMI bleeding events. Details of adjusted confounding factors were presented in supplementary Tables 3 and 4, respectively. The results expressed as a hazard ratio (HR) with a 95% CI. All the analyses were performed using software packages SPSS (version 25.0, IBM Corporation, New York, NY, USA) and GraphPad Prism 9.0. All statistical tests were two-sided and a value of *P* < 0.05 was considered significant.

## Results

From January 2017 to December 2019, 516 patients with CCS and AF who had undergone PCI and were indicated to use OAC based on CHA2DS2-VASc score ≥ 1, excluding sex, were included in the final analysis. The median follow-up time was 36 months (interquartile range:22–45).

Baseline characteristics, organized by the year of enrollment, were presented in Table 1. The average age was 66 ± 9 and 95 (18.4%) were female. Those enrolled in 2018 exhibited a slightly higher prevalence of hyperlipidemia and in 2019 a lower creatine clearance (CrCl) was observed. However, other demographic information and the history of comorbidities showed no difference among groups.

**Table 1:** Baseline characteristics according to enrolled years

Variables	2017 (*n* = 204)	2018 (*n* = 169)	2019 (*n* = 143)	Total (*n* = 516)	*P* value^*^
Age Female	66 ± 9 41 (20.1%)	65± 9 33 (19.5%)	67 ± 8 21 (14.7%)	66 ± 9 95 (18.4%)	0.246 0.397
BMI	25.97 ± 3.56	26.17 ± 3.25	25.73 ± 3.18	25.97 ± 3.36	0.526
SBP	131.81 ± 16.69	132.48 ± 17.52	134.43 ± 16.83	132.76 ± 17.01	0.357
DBP	78.07 ± 11.03	78.21 ± 12.25	77.53 ± 10.50	77.97 ± 11.29	0.858
LVEF	59.83 ± 7.73	58.50 ± 9.34	59.39 ± 8.05	59.25 ± 8.38	0.314
CrCl	81.75 (68.68–95.92)	79.57 (71.01–91.67)	74.85 (64.80-88.88)	79.36 (67.64–92.19)	0.010
HAb1c	6.1 (5.8-7.0)	6.2 (5.9-7.0)	6.3 (5.9–7.1)	6.2 (5.8-7.0)	0.202
Current smoker	52(25.5%)	37(21.9%)	25(17.5%)	114(22.1%)	0.208
HAS-BLED score ≥ 3	64(31.4%)	59(34.9%)	53(37.1%)	176(34.1%)	0.526
CHA2DS2-VASc score	3 (2–4)	3 (2–4)	3 (2–4)	3 (2–4)	0.642
** Medical history**					
MI	43(21.1%)	44(26.0%)	42(29.4%)	129(25.0%)	0.199
PCI	64(31.4%)	43(25.4%)	36(25.2%)	143(27.7%)	0.323
DM	86 (42.2%)	67 (39.6%)	59 (41.3%)	212 (41.1%)	0.885
HT	161 (78.9%)	140 (82.8%)	116 (81.1%)	417 (80.8%)	0.629
HF	33 (16.2%)	22 (13.0%)	28 (19.6%)	83 (16.1%)	0.290
CAD	156(76.5%)	123(72.8%)	96(67.1%)	375(72.7%)	0.158
PAD	32(15.7%)	28(16.6%)	23(16.1%)	83(16.1%)	0.974
CKD	10 (4.9%)	7 (4.1%)	6 (4.2%)	23 (4.5%)	0.924
CABG	11 (5.4%)	7 (4.1%)	12 (8.4%)	30 (5.8%)	0.264
TIA/stroke	52 (25.5%)	43 (25.4%)	37 (25.9%)	132 (25.6%)	0.996
Bleeding	13 (6.4%)	8 (4.7%)	7 (4.9%)	28 (5.4%)	0.744
Hyperlipidemia	152(74.5%)	141(83.4%)	102(71.3%)	395(76.6%)	0.029
Hyperthyroidism	20(9.8%)	13(7.7%)	9(6.3%)	42(8.1%)	0.484
** AF type**					0.660
New-onset AF	9(4.4%)	14(8.3%)	9(6.3%)	35(6.4%)	
PAF	118(57.8%)	95(56.2%)	82(57.3%)	295(57.2%)	
PeAF	77(37.7%)	60(35.5%)	52(36.4%)	189(36.6%)	
** Medication**					
Acid antisecretory	143 (70.1%)	104 (61.5%)	93 (65.0%)	340 (65.9%)	0.215
Statins	201 (98.5%)	167 (98.8%)	140 (97.9%)	508 (98.4%)	0.803
β-blockers	176 (86.3%)	148 (87.6%)	116 (81.1%)	440 (85.3%)	0.242
ACEI/ARB	129 (63.2%)	112 (66.3%)	92 (64.3%)	333 (64.5%)	0.829
CCB	84 (41.2%)	75 (44.4%)	64 (44.8%)	223 (43.2%)	0.749

Data are presented as number(proportion), mean ± SD or median (interquartile range)

BMI, body mass index; SBP, systolic blood pressure; DBP, diastolic blood pressure; LVEF, left ventricular ejection fraction; MI, myocardial infarction; PCI, percutaneous coronary intervention; DM, diabetes mellitus; HT, hypertension; HF, heart failure; CAD, stable coronary artery disease; PAD, peripheral arterial disease; CKD, chronic kidney disease; CABG, coronary artery bypass grafting; TIA, transient ischemic attack; CrCl, creatine clearance; HAb1c, glycosylated hemoglobin; AF, atrial fibrillation; PAF, paroxysmal atrial fibrillation; PeAF, persistent atrial fibrillation and permanent atrial fibrillation; ACEI: Angiotensin-Converting Enzyme Inhibitors; ARB: Angiotensin Receptor Blocker; CCB: Calcium Channel Blockers

^*^P value was based on χ^2^test, ANOVA and Kruskal-Wallis *H* test as appropriate

### Status of antithrombotic therapy

Table 2 presented the status of antithrombotic therapy according to the years of enrollment. The utilization of clopidogrel and ticagrelor remained stable across the years without any significant differences. The prescription of aspirin showed a slight decrease. There was a notable increase in the usage of OAC (*P* < 0.001), primarily attributed to the rising trend of NOAC usage. In contrast, the usage of warfarin showed no significant difference among the groups. DAT showed an increasing trend (*P* = 0.022). Similarly, there was a significant increase in the group received TAT (*P* < 0.001).

**Fig. 2:**
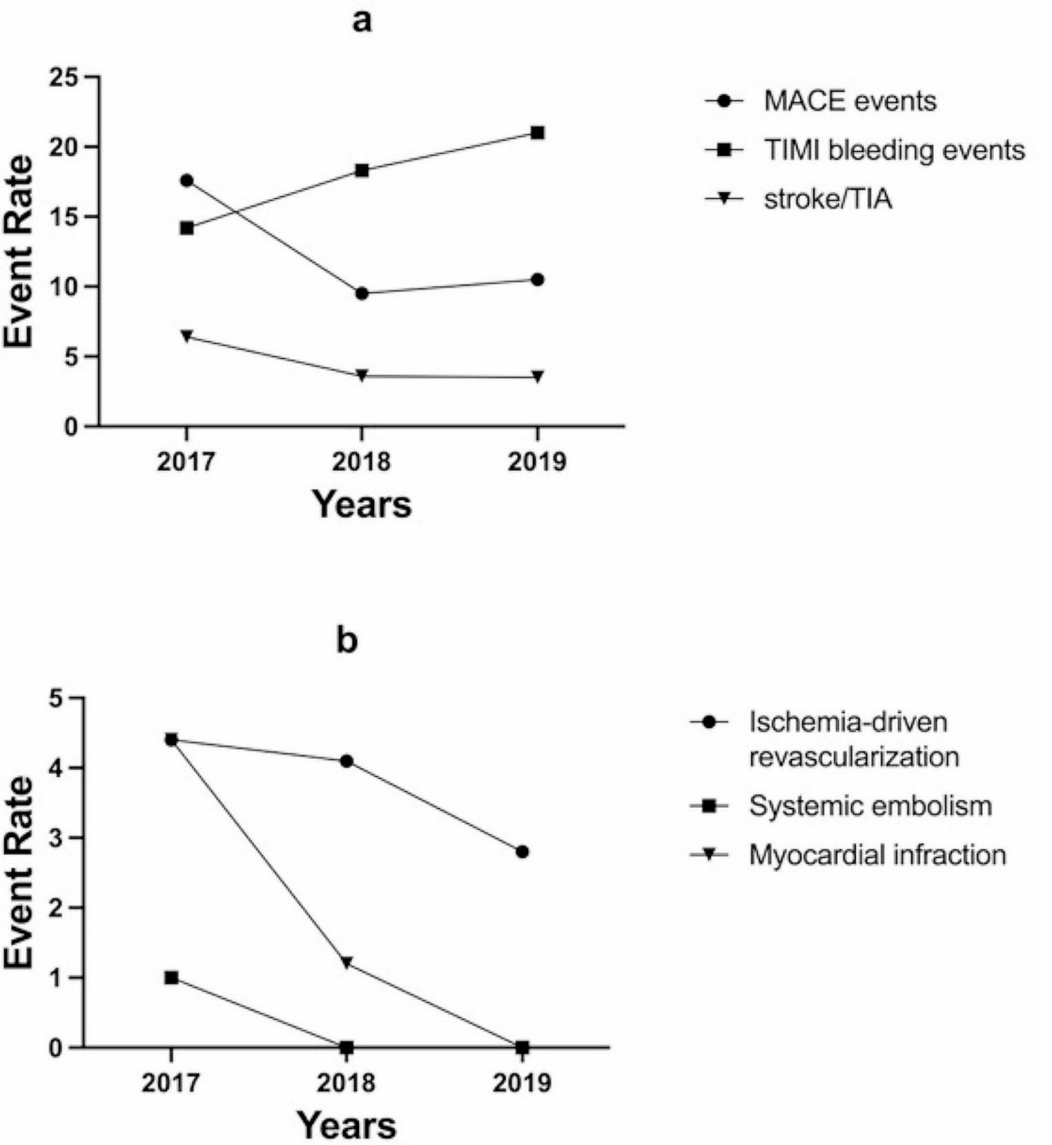
Tendency of event rate according to enrolled years. MACE events included all-cause death, myocardial infarction, stroke/transient ischemic attack (TIA), systemic embolism or ischemia-driven revascularization. TIMI bleeding events included minor, minimal and major TIMI bleeding events based on TIMI criteria. TIA, Transient ischemic attack

Figure 2 illustrated a decreasing trend in MACE events, stroke/TIA, MI and SE, while TIMI bleeding events showed an increasing pattern.

**Table 2:** Status of antithrombotic therapy according to years

	2017 (*n* = 204)	2018 (*n* = 169)	2019 (*n* = 143)	Total (*n* = 516)	*P* value^*^
ASA	198 (97.1%)	164 (97.0%)	131 (91.6%)	493 (95.5%)	0.027
Clopidogrel	188 (92.2%)	158 (93.5%)	131 (91.6%)	477 (92.4%)	0.806
Ticagrelor	16 (7.8%)	9 (5.3%)	11 (7.7%)	36 (7.0%)	0.589
**OAC usage**	49 (24.0%)	62 (36.7%)	79 (55.2%)	190 (36.8%)	< 0.001
VKA	22 (10.8%)	11 (6.5%)	15 (10.5%)	48 (9.3%)	0.311
NOAC	27 (55.1%)	51 (82.3%)	64 (81.0%)	142 (74.7%)	0.001
DAT	5 (2.5%)	7 (4.1%)	13 (9.1%)	25 (4.8%)	0.016
TAT	44 (21.6%)	55 (32.5%)	66 (46.2%)	165 (32.0%)	< 0.001

Data are presented as number(proportion)

ASA, aspirin; OAC, oral anticoagulant; VKA, warfarin; NOAC: non-vitamin K oral anticoagulant; DAT, double antithrombotic therapy; TAT, triple antithrombotic therapy

^*^P value was based on χ^2^test

### Subgroup analysis by antithrombotic therapy and OAC usage

The characteristics of patients according to antithrombotic therapy were presented in Table 3. Lower creatine clearance (CrCl, *P* = 0.023) was observed in TAT group. Those with lower left ventricular ejection fraction (LVEF) tended to be prescribed with TAT (*P* = 0.015). Besides, non-TAT was more likely to be utilized in patients with hyperlipidemia (*P* < 0.001). On the other hand, patients with diabetes mellitus (DM, *P* = 0.031) or heart failure (HF, *P* = 0.034) were more likely to be prescribed with TAT. Therefore, higher HAb1c (*P* = 0.007) was also observed in the TAT groups. In the comparison between the TAT and non-TAT groups, a higher BMI was associated with TAT therapy (*P* = 0.037).

**Fig. 3:**
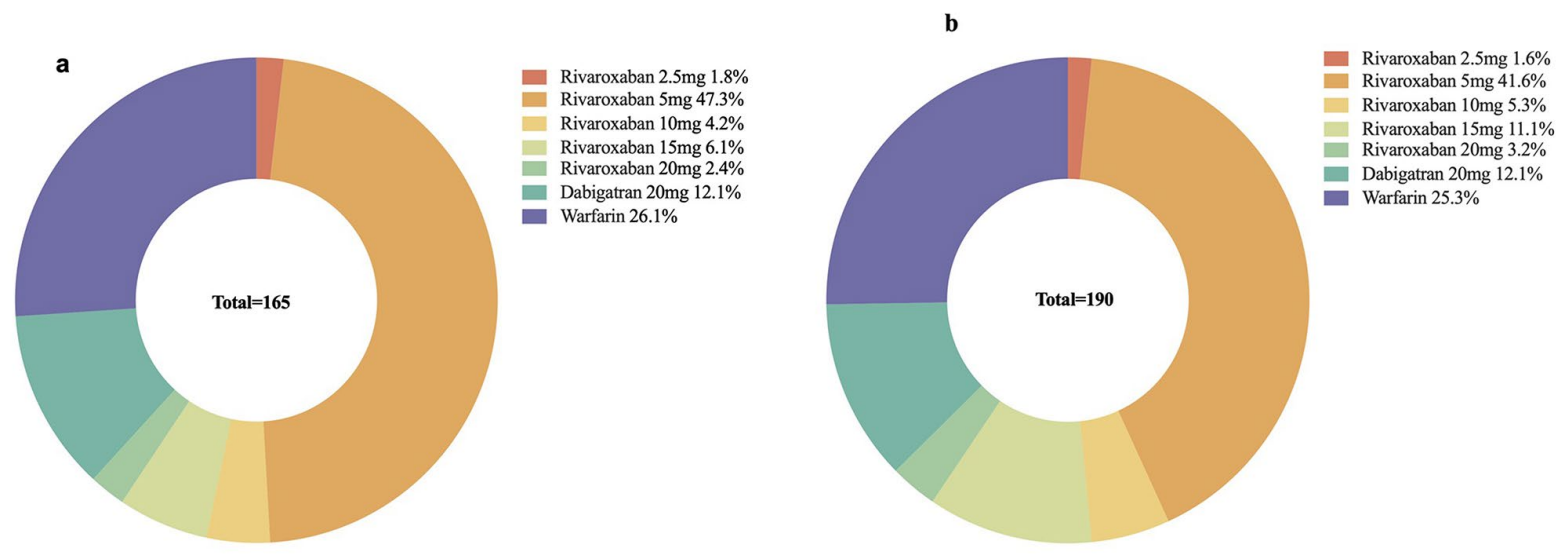
Proportion of oral anticoagulation (OAC) usage among patients prescribed with triple antithrombotic therapy (**a**) and combine therapy (**b**) based on OAC type and daily dose

Details of the proportion of OAC usage in patients prescribed with TAT were shown in Fig. 3, with rivaroxaban divided into five parts according to the daily prescription dose. Dabigatran was administered at 220 mg daily, and the dose of VKA was tailored for individual patients. Overall, only 46.7% used the standard dose for stroke prevention.

**Fig. 4:**
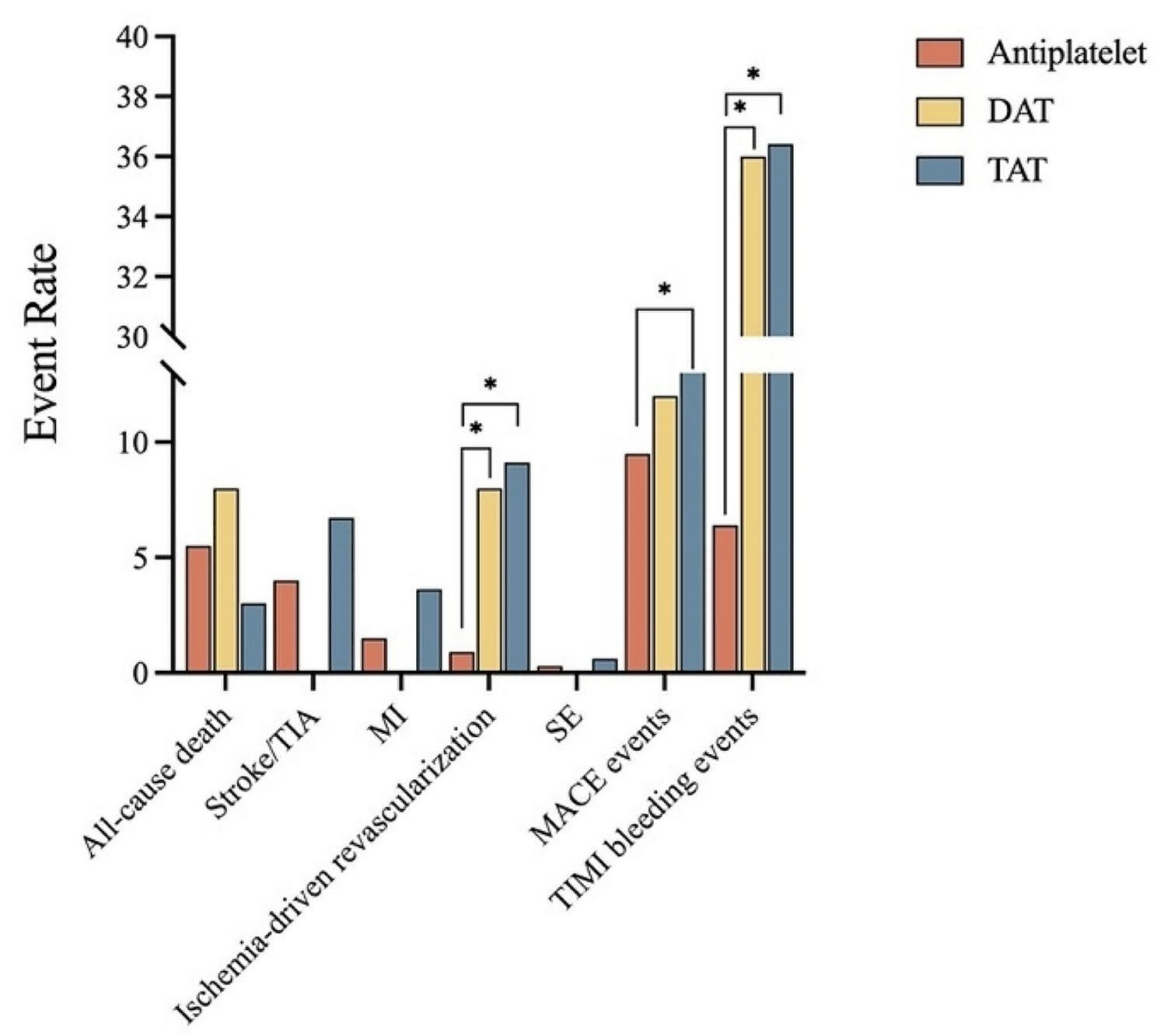
Event rate based on antithrombotic therapy. DAT, double antithrombotic therapy TAT, triple antithrombotic therapy; TIA, Transient ischemic attack; MI, myocardial infraction; SE: systemic embolism; MACE events included all-cause death, myocardial infarction, stroke, systemic embolism or ischemia-driven revascularization. TIMI bleeding events included minor, minimal and major TIMI bleeding events based on TIMI criteria. *P < 0.05, *P* value was based on χ^2^ test

Figure 4 depicted the percentage of events according to antithrombotic therapy during the follow-up period. MACE events showed differences between TAT and antiplatelet therapy. TIMI bleeding events presented differences between combined therapy (antiplatelet therapy plus OAC) and antiplatelet therapy. Similar results were detected in ischemia-driven revascularization.

As demonstrated in the supplementary Table 3, OAC therapy was more likely to be administered to patients with a history of DM (*P* = 0.010), a diagnosis with HF (*P* = 0.010), and less likely in those diagnosed with hyperlipidemia (*P* < 0.001) and previous PCI (*P* = 0.049). In addition, patients with PAF (*P* < 0.001) were favored of non-OAC treatment, while patients with peAF (*P* < 0.001) exhibited the opposite preference. Furthermore, peak cardiac troponin I (cTnI, *P* < 0.001) and N-terminal pro-B-type natriuretic peptide (NT-proBNP, *P* < 0.001) also presented an association with OAC usage.

**Fig. 5:**
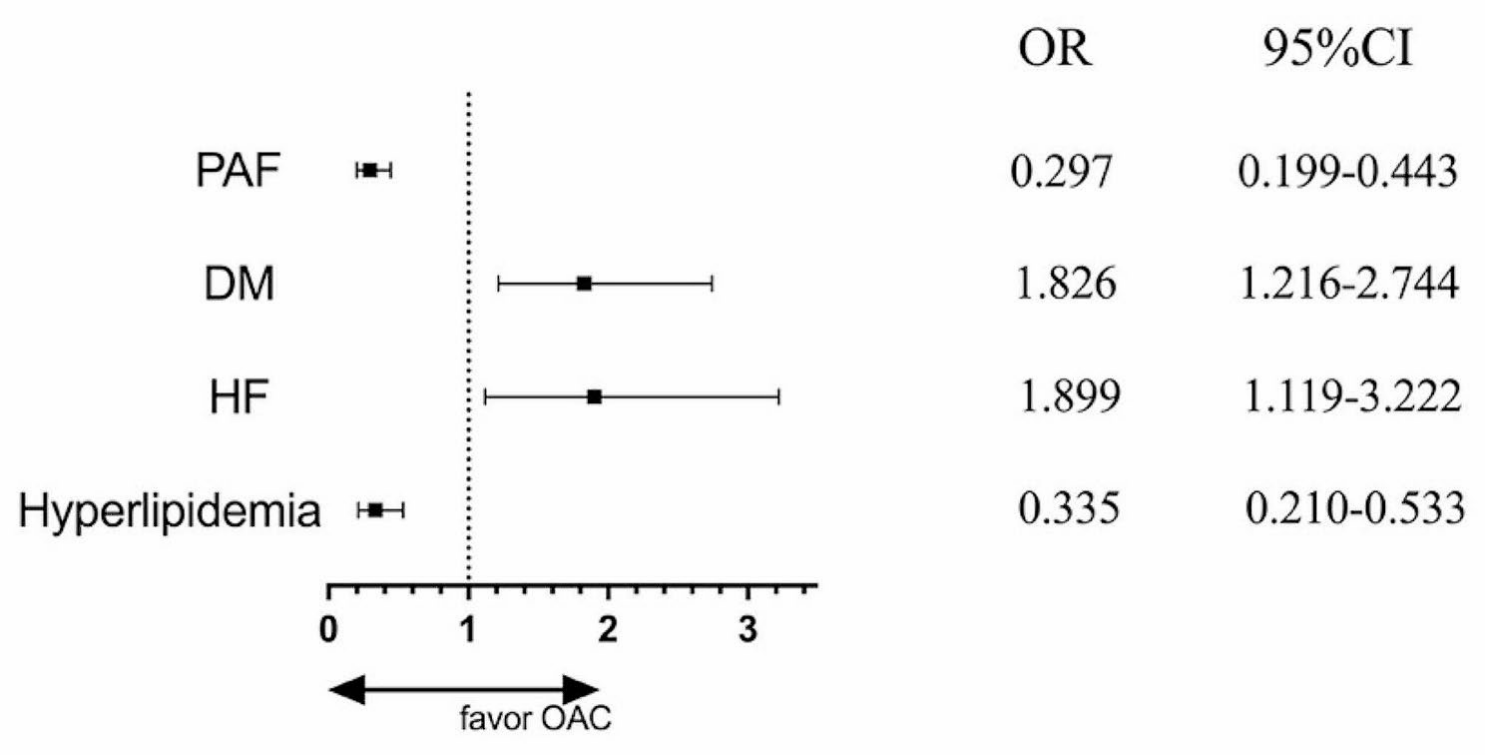
Predictors of the choice of oral anticoagulant (OAC) therapy. OR, odds ratio; CI, confidence interval; PAF, paroxysmal atrial fibrillation; DM, diabetes mellitus; HF, heart failure. Multiple logistic regression analysis adjusted history of heart failure, diabetes mellitus, hyperlipidemia, paroxysmal atrial fibrillation, PeAF which includes persist atrial fibrillation and permanent atrial fibrillation, creatinine clearance, left ventricular ejection fraction, glycosylated hemoglobin, peak cTnI, peak NT-proBNP

Subsequently, the multivariate logistic regression analysis was performed to assess independent factors capable of predicting the prescription of OAC therapy (Fig. 5). After adjustment for multivariate factors, peak cTnI, peak NT-proBNP and previous PCI showed no influence on OAC choice. However, patients with a history of DM (OR = 1.826; 95%CI, 1.216–2.744) and HF (OR = 1.899; 95%CI, 1.119–3.222) were favored of OAC therapy. Notably, HF emerged as the strongest predictor of OAC prescription. Conversely, hyperlipidemia (OR = 0.335; 95%CI, 0.210–0.533) and PAF (OR = 0.297; 95%CI, 0.199–0.443) were associated with a preference for non-OAC therapy.

**Table 3:** Characteristics of patients according to antithrombotic therapy regimen

	Non-TAT (*n* = 351)		TAT (*n* = 165)	*P* value^#^	*P* value^*^
	Antiplatelet (*n* = 326)	DAT (*n* = 25)			
Age	66 ± 9	70 ± 8	66 ± 8	0.479	0.105
Female	61 (18.7%)	8 (32.0%)	26 (15.8%)	0.286	0.145
BMI	25.81 ± 3.32	25.12 ± 3.76	26.42 ± 3.33	0.037	0.078
SBP	133.50 ± 17.57	133.88 ± 17.47	131.12 ± 15.73	0.118	0.502
DBP	78.37 ± 11.74	73.44 ± 10.77	77.87 ± 10.32	0.890	0.108
CrCl	80.96 (70.91–93.61)	74.85 (67.59–89.14)	76.03 (65.81–90.74)	0.023	0.034
LVEF	59.91 ± 7.80	59.16 ± 9.31	57.94 ± 9.20	0.015	0.047
HAb1c	6.16 (5.80-7.00)	6.20(5.90–7.05)	6.40(5.90–7.40)	0.007	0.024
**AF type**				< 0.001	< 0.001
New-onset	19 (5.8%)	1 (4.0%)	12 (7.3%)		
PAF	223 (68.4%)	14 (56.0%)	58 (35.2%)		
PeAF	84(25.8%)	10(40.0%)	95(57.6%)		
**Medical history**					
HT	257 (78.8%)	23 (92.0%)	137 (83.0%)	0.381	0.186
HF	42(12.9%)	5(20.0%)	36(21.8%)	0.015	0.034
DM	120 (36.8%)	11 (44.0%)	81 (49.1%)	0.011	0.031
Hyperlipidemia	273 (83.7%)	16 (64.0%)	106 (64.2%)	< 0.001	< 0.001
Stroke/TIA	76 (23.3%)	8 (32.0%)	48 (29.1%)	0.210	0.288
Bleeding	18(5.5%)	0(0.0%)	10(6.1%)	0.663	0.456
HAS-BLED score ≥ 3	81 (24.8%)	9 (36.0%)	50 (30.3%)	0.267	0.260
**Medication**					
Acid antisecretory	212 (65.0%)	18 (72.0%)	110 (66.7%)	0.799	0.753
Statins	320 (98.2%)	25 (100.0%)	163 (98.8%)	0.670	0.706
β-blockers	277 (85.0%)	19 (76.0%)	144 (87.3%)	0.379	0.323
ACEI/ARB	203 (62.3%)	17 (68.0%)	113 (68.5%)	0.198	0.370
CCB	132 (40.5%)	14 (56.0%)	77 (46.7%)	0.278	0.178

Data are presented as number(proportion), mean ± SD or median (interquartile range)

DAT, double antithrombotic therapy; TAT, triple antithrombotic therapy; BMI, body mass index; SBP, systolic blood pressure; DBP, diastolic blood pressure; CrCl, creatinine clearance; LVEF, left ventricular ejection fraction; HAb1c, glycosylated hemoglobin; HT, hypertension; HF, heart failure; DM, diabetes mellitus; PAF, paroxysm atrial fibrillation; PeAF includes persist atrial fibrillation and permanent atrial fibrillation; TIA, Transient ischemic attack; ACEI: Angiotensin-Converting Enzyme Inhibitors; ARB: Angiotensin Receptor Blocker; CCB: Calcium Channel Blockers

^#^*P* value was based on χ^2^ test, *t* test and Mann-Whitney *U* test as appropriate between non-TAT and TAT

^*^P value was based on χ^2^ test, ANOVA and Kruskal-Wallis *H* test as appropriate among antiplatelet, DAT and TAT

### Prognosis of patients with CCS and AF underwent PCI

Regarding the prognosis, we conducted separate analyses for MACE events and TIMI bleeding events, respectively. After adjusting for potentially confounding variables through multivariate Cox regression, a history of HF (HR, 1.744; 95%CI, 1.011–3.038) and TAT (HR, 2.708; 95%CI, 1.653–4.436) showed an independent association with MACE events, as detailed in Table 4.

In terms of bleeding events, there was a decrease in the risk of bleeding with an increase in CrCl (HR, 0.986; 95%CI, 0.974–0.997). On the other hand, OAC therapy (HR, 10.378; 95%CI, 6.136–17.555) emerged as a risk factor for TIMI bleeding events. (Table 5)

**Table 4:** Predictors with hazard ratio (HR) and 95%CI for MACE events

Variates	HR	95%CI	*P* value
HF	1.744	1.011–3.038	0.050
TAT	2.708	1.653–4.436	< 0.001

CI, confidential interval; HF, heart failure; TAT, triple antithrombotic therapy

MACE events included all-cause death, myocardial infarction, stroke/transient ischemic attack (TIA), systemic embolism or ischemia-driven revascularization

Multivariate cox regression adjusted the history of heart failure, the use of triple antithrombotic therapy and antiplatelet therapy

**Table 5:** Predictors with hazard ratio (HR) and 95%CI for TIMI bleeding events

Variates	HR	95%CI	*P* value
CrCl	0.986	0.974–0.997	0.014
OAC	10.378	6.136–17.555	< 0.001

CI, confidential interval; CrCl, creatine clearance; OAC, oral anticoagulant

TIMI bleeding events included minor, minimal and major TIMI bleeding events based on TIMI criteria

Multivariate cox regression adjusted history of hyperlipidemia, creatinine clearance, paroxysmal atrial fibrillation, PeAF which includes persist atrial fibrillation and permanent atrial fibrillation, antiplatelet therapy and oral anticoagulant therapy

## Discussion

In the present study, the principal findings could be summarized as followed, (a) The utilization of TAT therapy in patients with CCS and AF who underwent PCI had increased over years, as did the use of NOACs. (b) Anticoagulant therapy was underutilized in patients with CCS and AF, both in terms of limited usage rates and non-standard dosage. (c) Patients with a history of DM and HF were favored of OAC therapy, while patients with hyperlipidemia and PAF preferred non-OAC therapy. d)History of HF and TAT were independently associated with MACE events. Higher CrCl was protective factor for bleeding events, while OAC usage increased the risk of bleeding.

Anticoagulant therapy has been demonstrated to be effective in preventing stroke and SE among AF patients. However, antiplatelets have been proved no such benefits in recent decades [18]. The management of antithrombotic therapy in patients with PCI and AF is a challenging dilemma between bleeding and stroke risk and the choice between antiplatelet and anticoagulant [16, 17, 19]. A Japanese research indicated that the use of direct oral anticoagulants, rising from 15% in 2014 to 100% in 2018. Additionally, the utilization of TAT increased from approximately 10% to over 75% in 2018 in patients with AF undergoing PCI [20]. In our study, we observed an increase in the use of TAT as the antithrombotic regimen at the index of discharge from 2017 to 2019. The use of OACs, particularly NOACs, has also shown a significant increase over the years. Despite the recommendation to prescribe OAC based on the CHA2DS2-VASc score, a substantial number of patients were not receiving OACs. This was likely due to concerns about bleeding events in real-world scenarios. Although our study observed an increasing proportion of OAC therapy over the years, only 34.8% received OAC therapy, and 30.2% received TAT among our participants. Additionally, undertreated OAC therapy prescription was also noted in our study. The 2020 ACC Expert Consensus Decision recommended standard doses for rivaroxaban (20 mg once daily) or dabigatran (110 mg twice daily). Patients with CrCl ≤ 50 mL/min or those using adjunctive P2Y_12_ inhibitors were deemed suitable for a reduced dose of rivaroxaban (15 mg once daily) [21]. In our study, around half of patients prescribed with OAC were undertreated, potentially resulting in less effective prevention of TAT on MACE events. Therefore, there is still a need for improvement in antithrombotic therapy regimens.

A cohort included patients with non-valvular AF and a CHA2DS2-VASc score of 2 or more who were not receiving OAC therapy, suggesting evidence on patients of PAF. One of the top 5 reasons for non-OAC therapy in AF was low AF burden [22], aligning with our findings that patients with PAF were less likely to be treated with OAC. However, when CAD is considered as a cardiovascular risk factor in combination with AF, it is believed to lead to a worse prognosis [23]. KP-RHYTHM study investigated the relationship between AF burden and thromboembolism in PAF patients not receiving OAC therapy. This study demonstrated that a greater burden of PAF was associated with a significantly higher rate of thromboembolism [24], emphasizing the importance of OAC usage even in patients with PAF. Hyperlipidemia is commonly viewed as a risk factor for atherosclerosis [25]. Considering the potential for significant cardiovascular outcomes resulting from unstable plaque, practitioners were more inclined to select antiplatelet therapy [26]. This inclination may clarify our preference for non-OAC treatment in patients with hyperlipidemia. Furthermore, our limited data could have introduced bias in our evaluation of patients with hyperlipidemia. Nevertheless, hyperlipidemia has been proven to be a risk factor associated with unfavorably altered fibrin clot properties, linked to an increased cardiovascular risk, including stroke [27]. Despite the preference for non-OAC therapy in patients with PAF or hyperlipidemia, those conditions still pose a risk for stroke and should be evaluated carefully. On the contrary, consistent with our study, previous studies have also proved that HF and DM were predictors of ischemic stroke in AF patients [28–30], highlighting the essentiality of OAC usage in such cases.

The predictive model for MACE events in our study comprised a history of HF and TAT. Numerous studies have demonstrated that patients with both HF and AF face an increased risk of adverse events, including MACE events [31, 32]. Our study further confirmed that HF remained a predictor in patients with AF and CCS undergoing PCI, indicating an elevated risk of MACE events. Apart from the undertreatment with OAC, subgroup analysis revealed that patients with lower LVEF, a heavier burden of AF, a history of HF, and DM were more likely to be treated with TAT. These risk factors potentially leading to poorer prognosis.

An observational study conducted in Taiwan revealed that higher CrCl was associated with a lower trough concentration of OAC [33], contributing to bleeding prevention. Consistent with this perspective, our study also supported the notion that higher CrCl served as a protective factor against bleeding events.

Current clinical analyses involved with both ACS and CCS undergoing PCI [8, 34]. Although current guidelines recommending similar treatment regimens for those patients [7], ACS and CCS represented distinct cardiac risks [15]. It’s worth noting that this research focused on CCS patients undergoing PCI with AF. To our knowledge, this study was the first to describe the status of antithrombotic therapy specifically focusing on patients with CCS undergoing PCI.

Evidence on antithrombotic therapy and the prognosis of patients with AF undergoing PCI was limited in Asian population. Our study aimed to fill this gap by describing the changes in antithrombotic therapy over the years and identifying predictors affecting prognosis. Additionally, our study focused on patients whose CHA2DS2-VASc score was ≥ 1, excluding sex from the risk assessment. We selected patients based on this criterion, divided them according to realistic OAC regimens, and identified underlying factors influencing OAC usage. Our real-world analysis revealed that the percentage of OAC usage was still relatively low, highlighting the undertreated status. This issue should be addressed by enhancing awareness among physicians and patients regarding the importance of OAC usage in preventing stroke. Furthermore, the independent risk factors for MACE and TIMI bleeding events could help guide physicians in determining the balance between bleeding and ischemic events in the future for patients with CCS and AF who have undergone PCI. This may enable the provision of personalized antithrombotic therapy.

However, our study also has some limitations. Data were collected from a single center limiting the generalizability of our findings to the overall population of patients with CCS and AF undergoing PCI in China. Secondly, we didn’t involve patients’ over-time antithrombotic regimens and dynamic CHA2DS2-VASc scores over the follow-up period. Hence, future study should consider these dynamic changes of antithrombotic therapy and explore the possible antithrombotic therapy tailored to Asian patients. Long-term real-world follow-up, including the assessment of over-time antithrombotic therapy, is needed to guide future decision-making.

## Conclusion

In line with current guidelines, the percentage of patients using TAT therapy in those with CCS and AF undergoing PCI has increased, so has the usage of NOAC therapy in our study. Patients with a history of DM and HF were favored of OAC therapy, while patients with hyperlipidemia and PAF preferred non-OAC therapy. History of HF and TAT were independently associated with MACE events. Higher CrCl was a protective factor against bleeding events, and OAC therapy increased the risk of bleeding events.

### Electronic supplementary material

Below is the link to the electronic supplementary material.

Supplementary Material 1

## Data Availability

No datasets were generated or analysed during the current study.
