# Supplementary material for: Antithrombotic therapy at discharge and prognosis in patients with chronic coronary syndrome and atrial fibrillation who underwent PCI: a real-world study
Source: Thromb J. 2024 Jul 17;22:65. doi: 10.1186/s12959-024-00628-1 (PMC11253490; doi:10.1186/s12959-024-00628-1)
Supplement: Supplementary file 1 — Supplementary Material 1 [file 12959_2024_628_MOESM1_ESM.docx]

**Supplementary Material**

**Definitions:**

Invasive treatment consisted of PCI like bare-mental/drug eluting stent implantation, balloon angioplasty and percutaneous transluminal coronary angioplasty. Heart failure was defined based on prior symptomatic heart failure and/or left ventricular ejection fraction <40%. Hypertension was defined as a clinical record of hypertension or blood pressure over 140/90 mmHg. The history of prior CAD was defined as myocardial infarction (MI) or acute MI. Peripheral artery diseases were consisted of artery stenosis or occlusion. History of stroke was patients who had ischemic stroke or hemorrhage stroke. CHA2DS2-VASc scores include congestive heart failure, hypertension, diabetes, vascular disease, age 65–74 years, and sex category (female) with 1 point and 2 points for age ≥75 years or stroke. HAS-BLED scores were calculated with 1 point each for uncontrolled hypertension with systolic blood pressure over 160 mm Hg, abnormal renal function, abnormal hepatic function, previous ischemic or hemorrhagic stroke, bleeding history or predisposition, elderly (age > 65 years), concomitant use of antiplatelet or non-steroid anti-inflammatory drug and excessive alcohol intake per week, and labile international normalized ratio (INR) is applied for patients receiving warfarin with over 3 times INR measurements.

Supplementary figure 1: Proportion of antithrombotic therapy according to years


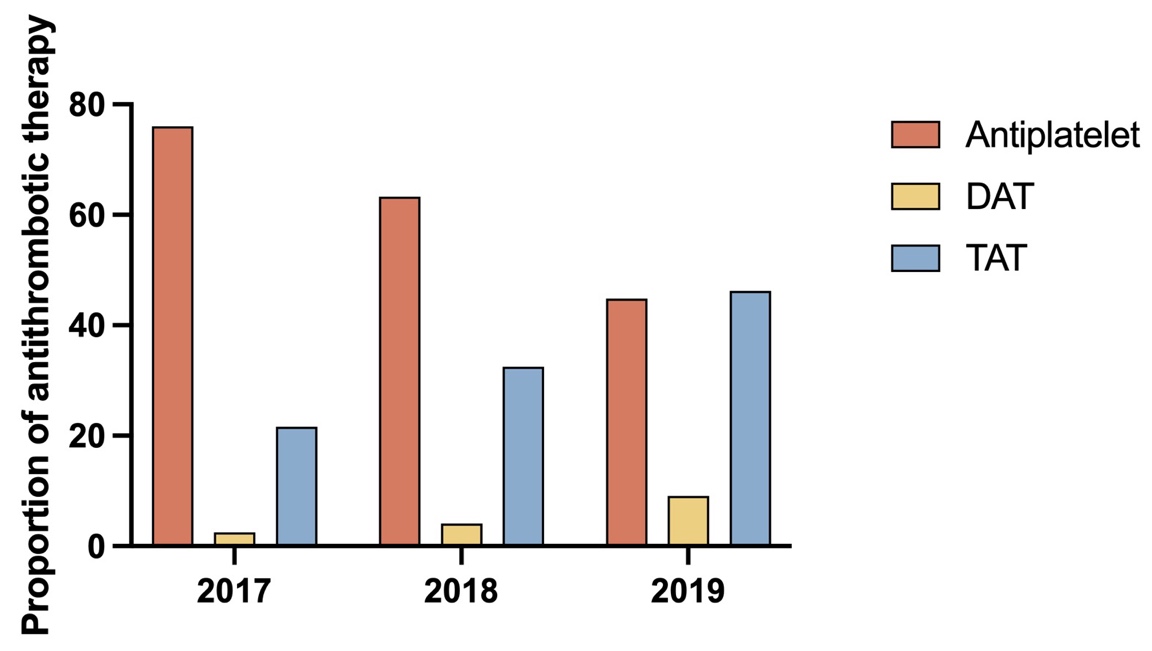


**DAT: dual antithrombotic therapy; TAT: triple antithrombotic therapy**

**Supplementary table 1 Rate of events according to enrolled year**

|  | 2017 | 2018 | 2019 | Total | *P* value^*^ |
| --- | --- | --- | --- | --- | --- |
| MACE | 36 (17.6%) | 16 (9.5%) | 15 (10.5%) | 67 (13.0%) | 0.038 |
| TIMI bleeding | 29 (14.2%) | 31 (18.3%) | 30 (21.0%) | 90 (17.4%) | 0.245 |
| Minimal bleeding | 0 (0.0%) | 1 (0.6%) | 0 (0.0%) | 1 (0.2%) | 0.605 |
| Minor bleeding | 27 (13.2%) | 25 (14.8%) | 29 (20.3%) | 81 (15.7%) | 0.191 |
| Major bleeding | 2 (1.0%) | 5 (3.0%) | 1 (0.7%) | 8 (1.6%) | 0.218 |
| MI | 9 (4.4%) | 2 (1.2%) | 0 (0.0%) | 11 (2.1%) | 0.011 |
| Ischemia-revascularization | 9 (4.4%) | 7 (4.1%) | 4 (2.8%) | 20 (3.9%) | 0.728 |
| SE | 2 (1.0%) | 0 (0.0%) | 0 (0.0%) | 2 (0.4%) | 0.339 |
| Stroke/TIA | 13 (6.4%) | 6 (3.6%) | 5 (3.5%) | 24 (4.7%) | 0.324 |
| Cardiovascular death | 10 (4.9%) | 3 (1.8%) | 4 (2.8%) | 17 (3.3%) | 0.224 |
| All-cause death | 14 (6.9%) | 4 (2.4%) | 7 (4.9%) | 25 (4.8%) | 0.132 |

**P* value was based on χ2 test

MACE events included all-cause death, myocardial infarction, stroke/transient ischemic attack (TIA), systemic embolism or ischemia-driven revascularization; MI, myocardial infraction; SE, systemic embolism

**Supplementary table 2 Rate of events according to antithrombotic therapy**

|  | Antiplatelet | DAT | TAT | Total | *P* value^*^ |
| --- | --- | --- | --- | --- | --- |
| MACE | 31 (9.5%) | 3 (12.0%) | 33 (13.0%) | 67 (13.0%) | 0.005 |
| TIMI bleeding | 21 (6.4%) | 9 (36.0%) | 60 (36.4%) | 90 (17.4%) | <0.001 |
| Minimal bleeding | 1 (0.3%) | 0 (0.0%) | 0 (0.0%) | 1 (0.2%) | 1.000 |
| Minor bleeding | 16 (4.9%) | 9 (36.0%) | 56 (33.9%) | 81 (15.7%) | <0.001 |
| Major bleeding | 4 (1.2%) | 0 (0.0%) | 4 (2.4%) | 8 (1.6%) | 0.632 |
| MI | 5 (1.5%) | 0 (0.0%) | 6 (3.6%) | 11 (2.1%) | 0.310 |
| Ischemia-revascularization | 3 (0.9%) | 2 (8.0%) | 15 (9.1%) | 20 (3.9%) | <0.001 |
| SE | 1 (0.3%) | 0 (0.0%) | 1 (0.6%) | 2 (0.4%) | 1.000 |
| Stroke/TIA | 13 (4.0%) | 0 (0.0%) | 11 (6.7%) | 24 (4.7%) | 0.217 |
| Cardiovascular death | 10 (3.1%) | 2 (8.0%) | 5 (3.0%) | 17 (3.3%) | 0.401 |
| All-cause death | 18 (5.5%) | 2 (8.0%) | 5 (3.0%) | 25 (4.8%) | 0.360 |

**P* value was based on χ2 test

Abbreviations were showed in table 2.

**Supplementary table 3 characteristics according to OAC**

|  | Non-anticoagulant therapy (n=326) | Anticoagulant therapy  (n=190) | *P* value |
| --- | --- | --- | --- |
| Age | 65.84±9.12 | 66.11±7.90 | 0.721 |
| Sex(female) | 61 (18.7%) | 34 (17.9%) | 0.817 |
| BMI | 25.91±3.32 | 26.25±3.41 | 0.125 |
| SBP | 133.50±17.57 | 131.48±15.95 | 0.181 |
| DBP | 78.37±11.74 | 77.28±10.46 | 0.295 |
| MI | 75 (23.0%) | 54 (28.4%) | 0.171 |
| PCI | 100 (30.7%) | 43 (22.6%) | 0.049 |
| CABG | 20 (6.1%) | 10 (5.3%) | 0.683 |
| HF | 42 (12.9%) | 41 (21.6%) | 0.010 |
| Hypertension | 257 (78.8%) | 160 (84.2%) | 0.135 |
| DM | 120 (36.8%) | 92 (48.4%) | 0.010 |
| TIA/stroke | 76 (23.3%) | 56 (29.5%) | 0.122 |
| CKD | 17 (5.2%) | 6 (3.2%) | 0.275 |
| Prior bleeding | 18 (5.5%) | 10 (5.3%) | 0.901 |
| Hyperlipidemia | 273 (83.7%) | 122 (64.2%) | <0.001 |
| LVEF | 59.91±7.80 | 58.10±9.20 | 0.023 |
| HAb1c | 6.50±1.090^a^ | 6.71±1.14 | 0.036 |
| CrCl | 83.23±20.49 | 78.93±18.94 | 0.018 |
| Peak NT-proBNP | 367.50（148.75-1147.88）^b^ | 786.10（282.33-1608.13）^c^ | <0.001 |
| Peak cTnI | 2.00（0.50-8.00）^d^ | 0.70（0.00-4.25） | <0.001 |
| **AF type** |  |  |  |
| New-onset AF | 19（5.8%） | 13（6.8%） | 0.645 |
| PAF | 223（68.4%） | 72（37.9%） | <0.001 |
| peAF | 84（25.8%） | 105（55.3%） | <0.001 |

a-2 missing patients; b-13 missing patients; c-8 missing patients; d-6 missing patients.

Data are presented as number(proportion), mean ± SD or median (interquartile range)

**P* value was based on χ2 test, t test and Mann-Whitney U test as appropriate.

BMI, body mass index; SBP, systolic blood pressure; DBP, diastolic blood pressure; MI; myocardial infarction; PCI; percutaneous coronary intervention; CKD: chronic kidney disease; CrCl, creatinine clearance; LVEF, left ventricular ejection fraction; HAb1c, glycosylated hemoglobin; HF, heart failure; DM, diabetes mellitus; PAF, paroxysm atrial fibrillation; PeAF includes persist atrial fibrillation and permanent atrial fibrillation; TIA, Transient ischemic attack

Variables with *P*<0.05 were entered into multiple logistic regression analysis model.

**Supplementary table 4 Univariate Cox proportional-hazards model performed for MACE events**

|  | HR | 95%CI | *P* value |
| --- | --- | --- | --- |
| Age | 1.019 | 0.991-1.049 | 0.19 |
| Sex | 1.061 | 0.579-1.945 | 0.847 |
| BMI | 1.016 | 0.947-1.090 | 0.665 |
| SBP | 1.002 | 0.988-1.016 | 0.802 |
| DBP | 1.010 | 0.989-1.031 | 0.352 |
| **Medical history** |  |  |  |
| HF | 1.973 | 1.137-3.423 | 0.016 |
| HT | 0.907 | 0.503-1.634 | 0.744 |
| Hyperlipidemia | 0.605 | 0.361-1.014 | 0.057 |
| DM | 1.343 | 0.831-2.169 | 0.228 |
| Stroke/TIA | 1.571 | 0.943-2.617 | 0.083 |
| bleeding | 1.799 | 0.778-4.164 | 0.170 |
| CKD | 1.765 | 0.709-4.390 | 0.222 |
| Current smoker | 1.494 | 0.885-2.522 | 0.133 |
| Creatine clearance | 0.992 | 0.979-1.004 | 0.187 |
| **AF type** |  |  |  |
| New-onset AF | 1.238 | 0.497-3.081 | 0.647 |
| PAF | 0.639 | 0.395-1.032 | 0.067 |
| PeAF | 1.579 | 0.971-2.566 | 0.066 |
| Antiplatelet | 0.353 | 0.215-0.578 | <0.001 |
| DAT | 1.167 | 0.365-3.728 | 0.794 |
| TAT | 2.842 | 1.741-4.640 | <0.001 |

Abbreviations were showed in Table S1.

Variables with *P*<0.05 were entered into multivariate model.

**Supplementary table 5 Univariate Cox proportional-hazards model performed for TIMI bleeding events**

|  | HR | 95%CI | *P* value |
| --- | --- | --- | --- |
| Age | 1.005 | 0.982-1.030 | 0.663 |
| Sex | 0.891 | 0.512-1.552 | 0.684 |
| BMI | 0.963 | 0.904-1.025 | 0.237 |
| SBP | 1.005 | 0.994-1.017 | 0.378 |
| DBP | 0.997 | 0.979-1.015 | 0.738 |
| **Medical history** |  |  |  |
| HF | 1.421 | 0.837-2.410 | 0.193 |
| HT | 1.138 | 0.663-1.954 | 0.639 |
| hyperlipidemia | 0.451 | 0.294-0.693 | 0.001 |
| DM | 1.036 | 0.682-1.574 | 0.868 |
| Stroke/TIA | 0.916 | 0.557-1.505 | 0.728 |
| Bleeding | 1.341 | 0.586-3.072 | 0.487 |
| CKD | 1.305 | 0.530-3.218 | 0.563 |
| Current smoker | 0.789 | 0.465-1.338 | 0.379 |
| CrCl | 0.981 | 0.069-0.992 | 0.001 |
| **AF type** |  |  |  |
| New-onset AF | 0.515 | 0.163-1.630 | 0.259 |
| PAF | 0.556 | 0.367-0.843 | 0.006 |
| PeAF | 2.084 | 1.379-3.152 | <0.001 |
| Antiplatelet | 0.092 | 0.55-0.155 | <0.001 |
| OAC | 10.857 | 6.433-18.324 | <0.001 |

Abbreviations were showed in Table S1.

Variables with *P*<0.05 were entered into multivariate model.
